# 403. Strain Epidemiology of Clostridioides difficile across Three Geographically Distinct Medical Centers in Chicago

**DOI:** 10.1093/ofid/ofac492.481

**Published:** 2022-12-15

**Authors:** Do Young Kim, Adam K Cheknis, Fidel Serna-Perez, Michael Y Lin, Mary K Hayden, Nicholas M Moore, Amanda Harrington, Vera Tesic, Kathleen G Beavis, Dale N Gerding, Stuart Johnson, Andrew M Skinner

**Affiliations:** University of Chicago, Chicago, Illinois; Edward Hines Jr. VA Hospital, Hines, Illinois; Edward Hines Jr., VA Hospital, Chicago, Illinois; Rush University Medical Center, Chicago, Illinois; Rush University Medical Center, Chicago, Illinois; Rush University Medical Center, Chicago, Illinois; Loyola University Chicago, Maywood, Illinois; University of Chicago, Department of Pathology, Chicago, Illinois; University of Chicago, Chicago, Illinois; Edward Hines, Jr. Veterans Affairs Hospital, Hines, Illinois; Hines VA Hospital and Loyola University Medical Center, Hines, Illinois; Loyola University Chicago Stritch School of Medicine, Maywood, Illinois

## Abstract

**Background:**

*Clostridioides difficile* infections (CDI) are caused by a large and diverse group of strains with differences in prevalence and associated morbidity. Over the past 20 years the *C. difficile* (CD) molecular epidemiology has changed as the prevalence of the epidemic strain recognized as restriction endonuclease analysis (REA) group BI or PCR-Ribotype group (RT) 027 has decreased. The objective of this study was to determine the current epidemiology of CD in the city of Chicago.

**Methods:**

Baseline characteristics and symptoms were compared for 81 patients who tested positive for CD by PCR (*tcdB*) between 9/1/2021 and 10/7/2021 at 3 hospitals in the city of Chicago. Patients were classified as having healthcare-associated CDI (HA-CDI) if symptoms began >72 hours after hospital admission, community-associated CDI (CA-CDI) if symptoms began ≤72 hours prior to admission, and community-onset healthcare-associated CDI (COHA-CDI) if they had been hospitalized ≤4 weeks prior to CDI diagnosis. Available stools were cultured and recovered CD isolates underwent REA typing. Determination of CD colonization was made by review of symptoms including chronicity of symptoms, stool frequency, and response to treatment.

**Results:**

Among all patients, 33% (27/81) were CA-CDI, 28% (23/81) COHA-CDI, 11% (9/81) HA-CDI, and 27% (22/81) were classified as colonized. Primary CDI accounted for 66% (39/59) of the infections. Among patients with a primary CDI, 46% (18/39) of patients were classified as CA-CDI whereas COHA-CDI and HA-CDI accounted for 54% (21/39) of infections. REA group Y was the most common group strain accounting for 29% (22/75) of isolates. (Figure 1) REA group Y accounted for 26% (7/27) of CA-CDI compared to 0 REA group BI [p=0.06], and REA group Y accounted for 35% (7/20) of all colonized patients. (Figure 2)

**Figure 1:**
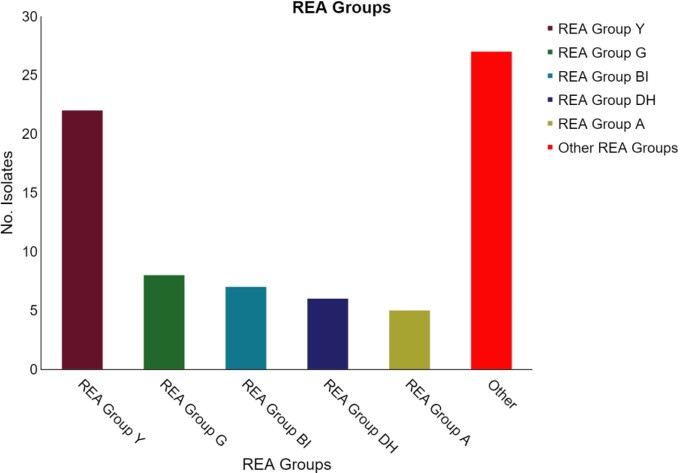

**Figure 2:**
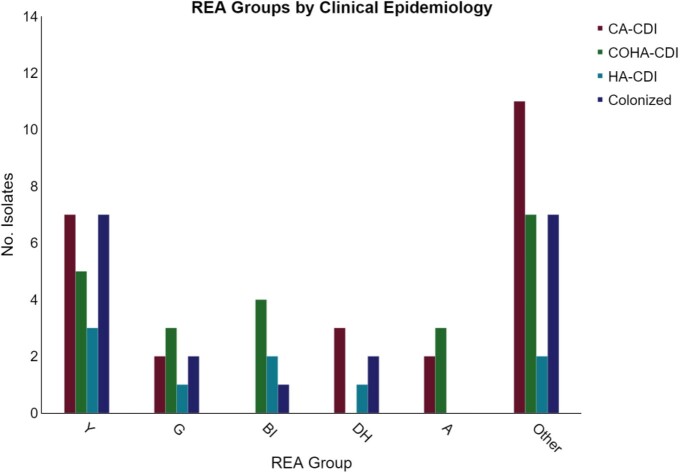

**Conclusion:**

There has been a marked change in the CD epidemiology within the city of Chicago since 2009 when REA group BI accounted for 61% of CDI (Black et al ICHE 2011; 32:897-902). REA group Y (typically identified as RT 014/020) is now the most common group strain in Chicago supplanting REA group BI (RT027). REA group Y appears to be associated primarily with CA-CDI and CD colonization. A detailed genomic analysis of REA group Y is required to determine potential reservoirs of REA group Y.

**Disclosures:**

**Mary K. Hayden, MD**, Sanofi: Member, clinical adjudication panel **Nicholas M. Moore, PhD, D(ABMM)**, Abbott Molecular: Grant/Research Support|Cepheid: Grant/Research Support **Amanda Harrington, PhD**, Beckman Coulter, Inc.: Clinical trial data collection funded by Beckman Coulter, Inc.|bioMeriuex/BioFire: Grant/Research Support **Dale N. Gerding, MD**, Destiny Pharma plc.: Advisor/Consultant **Stuart Johnson, M.D.**, Ferring Pharmaceuticals: Membership on Ferring Publication Steering Committee|Ferring Pharmaceuticals: Employee|Summit Plc: Advisor/Consultant.

